# Machine learning techniques based on ^18^F-FDG PET radiomics features of temporal regions for the classification of temporal lobe epilepsy patients from healthy controls

**DOI:** 10.3389/fneur.2024.1377538

**Published:** 2024-04-09

**Authors:** Kai Liao, Huanhua Wu, Yuanfang Jiang, Chenchen Dong, Hailing Zhou, Biao Wu, Yongjin Tang, Jian Gong, Weijian Ye, Youzhu Hu, Qiang Guo, Hao Xu

**Affiliations:** ^1^Department of Nuclear Medicine and PET/CT-MRI Center, Institute of Molecular and Functional Imaging, The First Affiliated Hospital of Jinan University, Jinan University, Guangzhou, China; ^2^The Affiliated Shunde Hospital of Jinan University, Foshan, Guangdong, China; ^3^Department of Radiology, Central People's Hospital of Zhanjiang, Zhanjiang, Guangdong, China; ^4^Epilepsy Center, Guangdong 999 Brain Hospital, Affiliated Brain Hospital of Jinan University, Guangzhou, Guangdong, China

**Keywords:** temporal lobe epilepsy, positron emission tomography (PET), radiomics, machine learning, ^18^F-FDG

## Abstract

**Background:**

This study aimed to investigate the clinical application of ^18^F-FDG PET radiomics features for temporal lobe epilepsy and to create PET radiomics-based machine learning models for differentiating temporal lobe epilepsy (TLE) patients from healthy controls.

**Methods:**

A total of 347 subjects who underwent ^18^F-FDG PET scans from March 2014 to January 2020 (234 TLE patients: 25.50 ± 8.89 years, 141 male patients and 93 female patients; and 113 controls: 27.59 ± 6.94 years, 48 male individuals and 65 female individuals) were allocated to the training (*n* = 248) and test (*n* = 99) sets. All 3D PET images were registered to the Montreal Neurological Institute template. PyRadiomics was used to extract radiomics features from the temporal regions segmented according to the Automated Anatomical Labeling (AAL) atlas. The least absolute shrinkage and selection operator (LASSO) and Boruta algorithms were applied to select the radiomics features significantly associated with TLE. Eleven machine-learning algorithms were used to establish models and to select the best model in the training set.

**Results:**

The final radiomics features (*n* = 7) used for model training were selected through the combinations of the LASSO and the Boruta algorithms with cross-validation. All data were randomly divided into a training set (*n* = 248) and a testing set (*n* = 99). Among 11 machine-learning algorithms, the logistic regression (AUC 0.984, F1-Score 0.959) model performed the best in the training set. Then, we deployed the corresponding online website version (https://wane199.shinyapps.io/TLE_Classification/), showing the details of the LR model for convenience. The AUCs of the tuned logistic regression model in the training and test sets were 0.981 and 0.957, respectively. Furthermore, the calibration curves demonstrated satisfactory alignment (visually assessed) for identifying the TLE patients.

**Conclusion:**

The radiomics model from temporal regions can be a potential method for distinguishing TLE. Machine learning-based diagnosis of TLE from preoperative FDG PET images could serve as a useful preoperative diagnostic tool.

## Introduction

Epilepsy, caused by abnormal discharge of brain neurons, is one of the most common chronic neurological disorders (1). Although multiple antiseizure drugs have been used to control seizures, drug resistance can still occur in approximately one-third of individuals with epilepsy. Surgical resection of the epilepsy focus is the best choice for individuals with drug-resistant epilepsy (2). Temporal lobe epilepsy (TLE) is the most common form of drug-resistant epilepsy, incurring considerable healthcare burdens (3, 4). Neuroimaging is a crucial clinical examination for epilepsy, and multiple imaging techniques, including brain MRI (structural, diffusion, and functional MRI) and PET, have been used to delineate abnormal brain structures and functions, which provides massive imaging data that can be analyzed by machine learning techniques for the identification of patients, localization of epilepsy focus, and prediction of medical and surgical outcomes (5, 6). Machine learning applications used for the differentiation of individuals with TLE and healthy subjects are mainly from MRI data. Various machine learning classifiers based on different MRI modalities, including T1, T2, DTI, DKI, and fMRI sequences, have achieved over 70–80% accuracy in successfully discriminating between patients with TLE and healthy controls (7–14). A study using the support vector machine method on independent components from rsfMRI data of 42 individuals with TLE achieved over 90% accuracy (15). In addition to MRI data, [18F]fluorodeoxyglucose (^18^F-FDG) PET imaging often indicates glucose hypometabolism in the epilepsy focus. Previous clinical PET applications were typically conducted in single conventional parameters for the differentiation of individuals with epilepsy and healthy subjects (16).

As an essential part of artificial intelligence, machine learning (ML) bridges statistics and computer science to learn relationships from data by developing efficient computing algorithms (17). In particular, a number of studies about ML techniques for imaging data analysis have achieved gratifying results. Quantitative, high-throughput data can be extracted, processed, and analyzed using machine learning techniques to discover associations with meaningful and hidden information that is inaccessible when using traditional approaches (18). Radiomics represents a burgeoning technique for image analysis that leverages algorithms or statistical tools to discern unique phenotypic variations in diseases from diagnostic imaging data. Subsequently, we investigated the possible differences in FDG PET radiomics features among TLE patients utilizing machine learning approaches. These characteristics were then compared with those of a control group comprising healthy subjects with the objective of evaluating their effectiveness in distinguishing patients with TLE from those in the control group.

## Materials and methods

### Participants

According to the criteria of the international league against epilepsy, 628 patients of diagnosed temporal lobe epilepsy who received brain ^18^F-FDG PET/CT examinations between March 2014 and January 2020 were retrospectively reviewed. Of the 628 TLE patients, we excluded 394 patients due to incomplete PET/CT data, unclear diagnosis, and postsurgical PET/CT data. Therefore, we only used data from the remaining 234 TLE patients (age = 25.50 ± 8.89 years, 141 male patients and 93 female patients) for our current study. For the control group, 113 age-matched controls (age = 27.59 ± 6.94 years, 48 male individuals and 65 female individuals) with extracranial lymphoma were reviewed. All controls had no history of neurological disorders, psychiatric conditions, chemotherapy, or radiotherapy. In short, an institutional cohort of 347 subjects (234 TLE patients and 113 HCs) was assessed in this study, which was divided into a training set for classification and a test set for validation. The study was approved by the local ethics committee of the First Affiliated Hospital of Jinan University and complied with the national legislation and the Declaration of Helsinki guidelines. All experimental protocols involving humans were performed in accordance with the guidelines set by national and international institutions. All participants provided consent to use their ^18^F-FDG PET results and clinical data for this study.

### PET/CT examinations

Each participant fasted for at least 6 h, and no clinical or EEG evidence of seizure onset was recorded for at least 2 h before ^18^F-FDG administration. PET/CT images were acquired in the 10-min static acquisition mode 50–70 min after injecting ^18^F-FDG intravenously at a dose of 0.08–0.10 mCi/kg body weight. Each participant was required to rest in a dimly lit and quiet room and was instructed to avoid reading with their eyes for approximately 30 min. The scanning range covered the whole brain. PET data were acquired in the 3D time-of-flight (TOF) mode in a one-bed position, with the overlap of 23.4%, a slice thickness of 3.27 mm, a slice interval of 3.75 mm, a pixel size of 3.64 mm, a matrix size of 192 × 192, and a scan field-of-view of 70 cm. The PET data were reconstructed in terms of the point spread function (PSF) together with TOF technology.

### Image preprocessing, radiomics feature extraction, and selection

For the 347 participants (234 individuals with temporal lobe epilepsy and 113 controls) included in our investigation, PET images underwent a series of preprocessing steps. Initially, Digital Imaging and Communications in Medicine (DICOM) data were converted to the Neuroimaging Informatics Technology Initiative (NIfTI) format following the Brain Imaging Data Structure (BIDS) guidelines, ensuring efficient management of neuroimaging datasets. Subsequently, to enhance the reproducibility of our analyses, PET images were coregistered to the Montreal Neurological Institute (MNI) standard space using the Advanced Normalization Tools (ANT) (version 0.3.2; https://github.com/ANTsX/ANTsPy) module—a Python (3.7.13) library optimized and validated for medical imaging applications. The ANTsPy module implements the ANTs registration method, offering both full and simplified interfaces for registering image pairs. The registration process employed a symmetric normalization transform, utilizing an affine, deformable transformation with mutual information as the optimization metric, and refined it for registrations with an additional affine step. After co-registration, each participant's PET image was reoriented to a standardized 182 × 218 × 182 voxel grid with 1.0 mm cubic voxels. This grid orientation ensured alignment of the subject's anterior-posterior axis parallel to the AC-PC line. By directly co-registering the original raw image data to a standardized space in a single step, a single interpolation of the image data was performed, minimizing resolution degradation and maintaining consistency across all scans. The PET images presented have undergone a smoothing procedure after the initial processing. Each set of images has been subjected to filtration utilizing a scanner-specific filter function, which may include non-isotropic filters, to generate images with a consistent isotropic resolution of 10 mm FWHM. Consequently, the lateral surgical temporal lobe regions were manually delineated slice by slice, aligning with the anatomical structure specified in the AAL atlas within the consistent MNI standard space, establishing them as 3D regions of interest (ROI). After this process, the lateral surgical temporal lobe regions were manually delineated slice by slice, aligning with the anatomical structure specified in the AAL atlas within the consistent MNI standard space, establishing them as 3D regions of interest (ROIs). We finally reviewed all PET images and the derived ROIs and found a perfect match. Each ROI on PET images was used for extracting radiomics features by an open-source package (PyRadiomics, version 3.0.1) (19). Prior to feature extraction, the PET images underwent z-score normalization, gray-level discretization with bin widths of 5 and voxel size resampling to 2 × 2 × 2 mm, utilizing PyRadiomics. The ideal number of bins for image discretization is defined in the order of 16–128 bins with a fixed bin width of 5. After processing all radiomics features by wavelet and Laplacian of Gaussian (LoG) filter methods, 1,132 features were extracted from each image sequence (original: 100 features, wavelet: 688 features, LoG: 344 features) (20, 21). In short, the intensity of glucose metabolic, shape, and textural features, or second-order features were calculated from the first-order statistics, geometrical statistics, the gray level co-occurrence matrix (GLCM), and gray level run-length matrix (GLRLM). The definitions of the features as well as the image pre-processing steps are explained in detail in the IBSI documentation.

Since high-dimensional data suffer from noise and redundant attributes that may weaken the performance of model training, multivariate logistic regression with the least absolute shrinkage and selection operator (LASSO) and Boruta algorithms with cross-validation were applied to reduce the high dimensionality of features and to select the radiomics features significantly associated with TLE (22). To discern crucial features from less significant ones, we initially utilized the Boruta feature selection algorithm to identify pertinent attributes within the training dataset. It is pertinent to highlight that the algorithm not only isolates relevant features but also establishes a hierarchical order of their significance. The Boruta algorithm leverages random forests to estimate feature relevance. As one of the classic algorithms of machine learning, random forest does classification or regression by combining the voting results of multiple decision trees. Furthermore, recognizing the remarkable feature selection prowess of the LASSO, we conducted LASSO regression alongside a comparative assessment, with LASSO operating on a regression analysis technique integrating feature selection and regularization concurrently, incorporating an L1 norm penalty within the minimization of the residual sum of squares. As the lambda parameter reaches a sufficient magnitude, certain coefficients can be effectively shrunk to zero. In this study, the tuning parameter λ (0.039) representing one standard error from the minimum was identified through cross-validation. The common features in the training set derived from the two methods were finally used for model establishment (23). The detailed results in the training set of the LASSO and Boruta algorithms are provided in the Supplementary material.

### Machine learning and model performance evaluation

In classification tasks of detecting TLE, 11 different machine learning algorithms were used in the training set: logistic regression (LR), Naive Bayes (NB), linear discriminant analysis (LDA), random forest (RF), Extra Trees Classifier (ET), Gradient Boosting Classifier (GBC), Light Gradient Boosting Machine (LBM), K-Nearest Neighbors (KNN), AdaBoost, Quadratic Discriminant Analysis (QDA), and Decision tree (DT). During the model training, the cross-entropy cost function was used to adapt weights during the learning process by minimizing the loss; and the optimal model hyperparameters were tuned by grid search algorithm and evaluated using 10-fold cross-validation. In addition, to fix the group imbalance in our dataset, the best way could be to generate additional samples for minority classes, which means that the model performance to correctly predict the minority class label is getting better by using SMOTE-ENN to balance our data (24). The SMOTE-ENN combination resampling method strategically integrates the advantages of the SMOTE technique and the Edited Nearest Neighbor (ENN) algorithm. By utilizing SMOTE to synthetically create new samples for mitigating data imbalance, followed by ENN to eliminate instances with discrepant class labels from their nearest neighbors, this method adeptly addresses imbalanced data sets while filtering out noise. Empirical evidence across diverse studies attests to its efficacy in augmenting classification prediction accuracy, positioning it as a promising solution for managing imbalanced class data in conjunction with classification algorithms (25).

**Figure 1:**
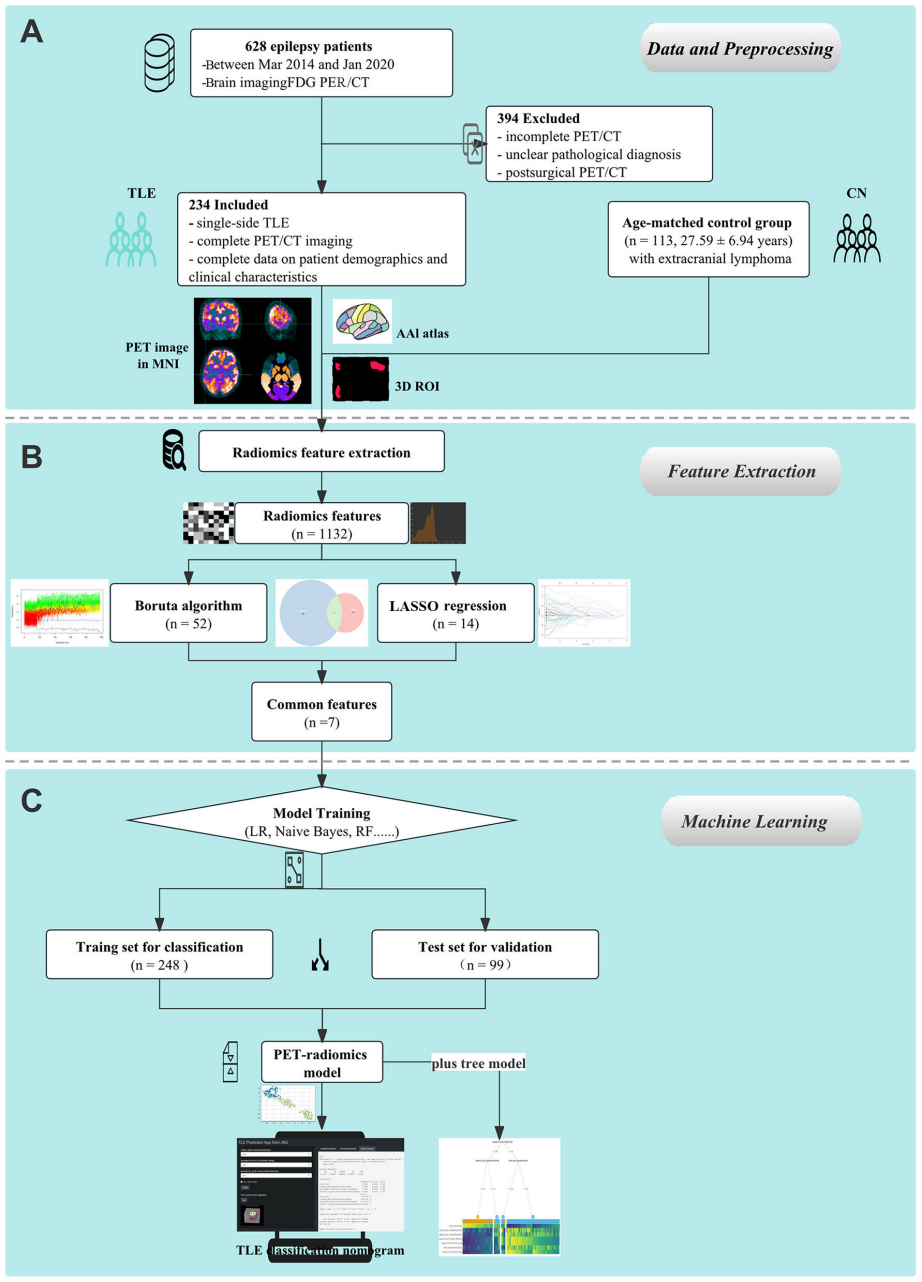
Schematic representation of the study's design and procedural steps. **(A)** Data collection and preprocessing on ^18^F-FDG PET images were performed. **(B)** PET radiomics features were extracted. **(C)** Machine learning models were established and compared.

After model training, each model was independently evaluated in the validation set to identify the TLE patients using area under the curve (AUC), accuracy, recall, precision, F1-score, Kappa, Matthews correlation coefficient (MCC), and Average precision score (APC). Based on the performance of radiomics classification model in the training set, the best model was tested in a validation set. The feature selection and classification methods were computed using Python 3. The threshold for statistical significance was set at a *p* < 0.05. The overall process is shown in Figure 1.

### Statistical analysis

For univariate analysis of data, the two-sided group t-test or the Mann–Whitney U test was used for continuous variables depending on the normality of the data, and the distributed measures were expressed as mean ± SD or median (25th percentile−75th percentile). A chi-squared test was performed for categorical variables, expressed as frequencies (rates). In this study, the statistical analysis was performed using software packages R (version 3.5.1; R Foundation for Statistical Computing, Vienna, Austria) and Python (version 3.7.13), and *p*-values of < 0.05 were considered statistically significant.

## Results

### Participants characteristics

Research participants included 234 TLE patients and 113 controls. Of all enrolled participants,248 of them were included in training the ML model, while the remaining 99 were included for testing the ML models. The mean age of the patient cohort was 25.50 years, with a mean onset time of 13.65 years and a mean duration time of 11.85 years. The clinical characteristics of the participants in the study cohort are summarized in Table 1. There were no differences in the clinical characteristics between the training and the test sets.

**Table 1:** Baseline characteristics of the datasets.

**Clinical characteristics**	**TLE patients (*n =* 234)**	**Healthy controls (*n =* 113)**
Age (years)	25.50 ± 8.89	27.59 ± 6.94
Age of onset (years, mean ± SD)	13.65 ± 9.73	-
Duration of epilepsy (years, mean ± SD)	11.85 ± 7.71	-
**Sex (** ***N*** **, %)**		
Female	93 (39.7)	65 (57.5)
Male	141 (60.3)	48 (42.5)
**Laterality (** ***N*** **, %)**		
Right	99 (42.3)	-
Left	135 (57.7)	-

SD, standard deviation.

### Feature extraction and selection

**Figure 2:**
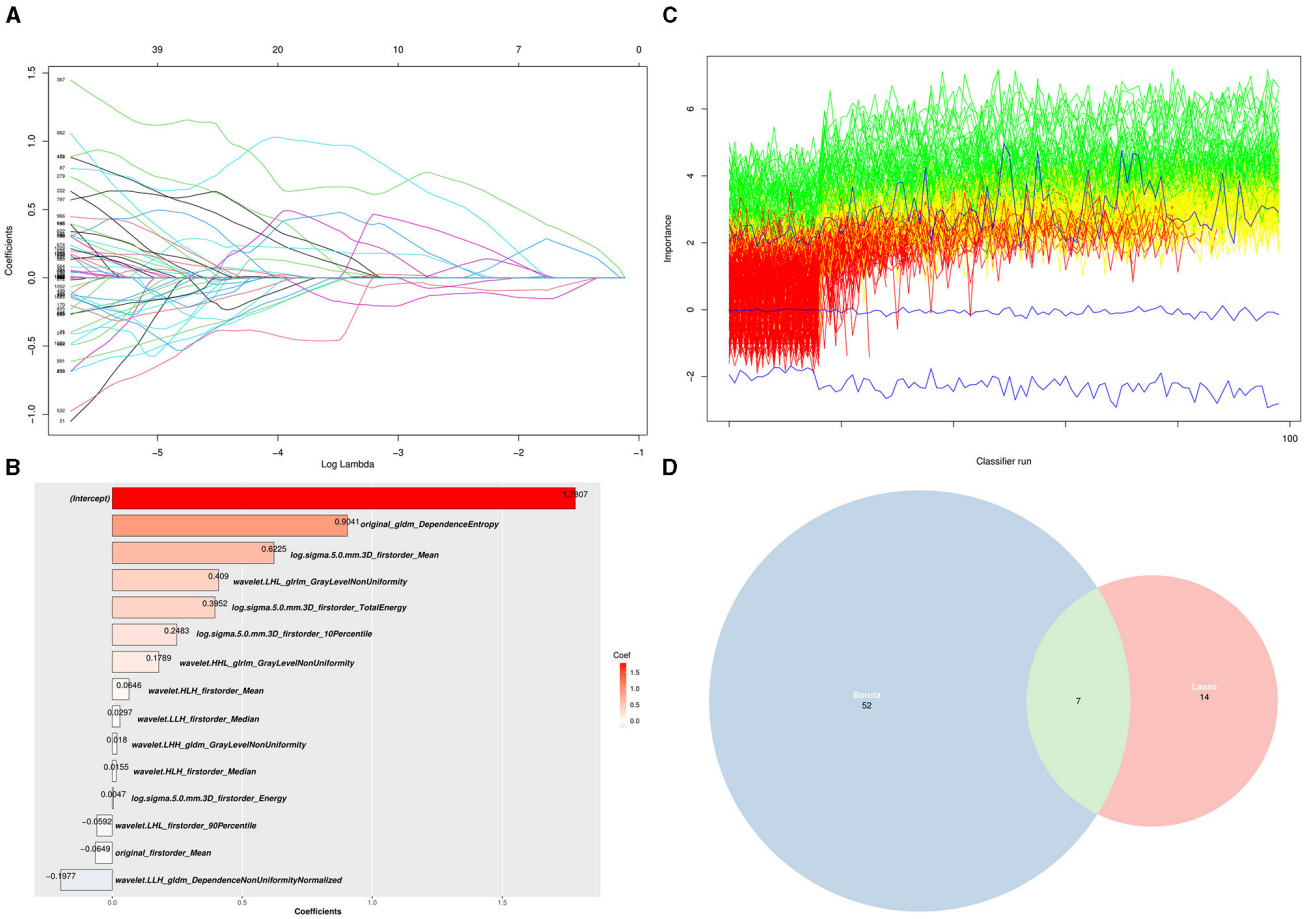
Selection of PET radiomics features using LASSO regression and Boruta algorithm. **(A)** LASSO regression analysis (each curve represents each PET radiomics features in the construction of the classification model). **(B)** The coefficients of 14 features selected by LASSO regression. **(C)** A plot method for Boruta objects, showing the attribute importance over run. Green lines correspond to confirmed attributes, red indicates rejected attributes, and blue indicates the importance of minimum, average, and maximum shaded attributes. **(D)** Seven shared target radiomics features among the two LASSO (52) and Boruta (14) algorithms.

A total of 1,132 radiomics features were extracted from each ROI of PET imaging, including 100 features from original data, 344 features filtered by the Laplacian of Gaussian method, and 688 features by the wavelet method. The LASSO regression and Boruta algorithms were used for feature selection, and the algorithms reduced the number of features to 14 (Figures 2A, B) and 52 (Figure 2C), respectively, resulting in seven common features to generate the machine learning model (Figure 2D).

### TLE prediction model training and validation

A cross-validation strategy was used to evaluate the performance of the ML models in the training set, mainly by the average area under the ROC curve (AUC). In total, 11 ML models, including LR, NB, LDA, RF, ET, GBC, LBM, KN, AdaBoost, QDA, and DT, were evaluated with cross-validated AUC of 0.984, 0.977, 0.977, 0.977, 0.977, 0.973, 0.973, 0.969, 0.947, 0.905, 0.878, respectively. The LR model obtaining the highest AUC for distinguishing the TLE patients and healthy controls was further validated. The accuracy, recall, precision, F1-score, Kappa, MCC, and APC of the LR model in the training set were 0.948, 0.941, 0.985, 0.959, 0.889, 0.899, and 0.994, respectively. The results of some other subject-level evaluation indexes of these models are shown in Table 2.

**Table 2:** Performance of 11 machine learning algorithms in the training set.

**Model**	**Accuracy**	**AUC**	**Recall**	**Prec**.	**F1**	**Kappa**	**MCC**	**APC**
Logistic regression	0.948	0.984	0.941	0.985	0.959	0.889	0.899	0.994
Naive Bayes	0.913	0.977	0.898	0.977	0.931	0.81	0.826	0.991
Linear discriminant analysis	0.936	0.977	0.924	0.984	0.949	0.862	0.873	0.992
Random forest classifier	0.918	0.977	0.924	0.962	0.938	0.816	0.829	0.992
Extra trees classifier	0.936	0.977	0.924	0.984	0.949	0.862	0.873	0.993
Gradient boosting classifier	0.878	0.973	0.907	0.922	0.909	0.716	0.732	0.99
Light gradient boosting machine	0.889	0.973	0.924	0.922	0.919	0.737	0.749	0.99
K neighbors classifier	0.936	0.969	0.924	0.985	0.949	0.864	0.877	0.985
Ada boost classifier	0.866	0.947	0.899	0.915	0.901	0.684	0.704	0.981
Quadratic discriminant analysis	0.803	0.905	0.832	0.898	0.846	0.539	0.566	0.947
Decision tree classifier	0.889	0.878	0.907	0.938	0.918	0.746	0.761	0.913

AUC, Area under ROC curve; Prec., Precision; MCC, Matthews correlation coefficient; APC, Average precision score.

### PET-radiomics model tuning and decision tree model validation

To further improve the predictability of the LR model, a tuned LR model was chosen by Akaike Information Criterion (AIC) in a stepwise algorithm. The results of seven radiomics features of the training set in a stepwise algorithm are shown in Table 3, then original_gldm_DependenceEntrop (OR = 7.14), log.sigma.5.0.mm.3D_firstorder_Energy (OR = 85.98), and wavelet.LHL_glrlm_GrayLevelNonUniformity (OR = 8.15) were ultimately used to generate the tuned PET-radiomics model. **Figure 4** shows the performance of the tuned LR models in the training and the test sets, with the AUCs of 0.981 and 0.957, respectively.

**Table 3:** The OR value of seven radiomics features of the LR model.

**Features**	**Univariate analysis**		**Multivariate analysis**	
	**OR (95 % CI)**	***p*-value**	**OR (95 % CI)**	***p*-value**
original_firstorder_Mean	0.01 (0.00–0.04)	< 0.001	-	
original_gldm_DependenceEntropy	27.47 (11.35–66.47)	< 0.001	7.14 (2.22–22.95)	0.001
log.sigma.5.0.mm.3D_firstorder_Energy	1894.65 (214.64–16723.87)	< 0.001	85.98 (5.54–1334.09)	0.002
log.sigma.5.0.mm.3D_firstorder_Mean	39.96 (15.40–103.70)	< 0.001	-	
log.sigma.5.0.mm.3D_firstorder_TotalEnergy	1894.65 (214.64–16723.87)	< 0.001	-	
wavelet.LHL_glrlm_GrayLevelNonUniformity	37.65 (14.88–95.27)	< 0.001	8.15 (2.78–23.91)	< 0.001
wavelet.LHH_gldm_GrayLevelNonUniformity	5.84 (3.58–9.50)	< 0.001	-	

OR, Odds ratio; CI, Confidence interval.

The results of seven radiomics features of the test set in the decision tree model are shown in Supplementary Figure S2. Log.sigma.5.0.mm.3D_firstorder_Mean was the root, while wavelet.LHL_glrlm_GrayLevelNonUniformity and original_gldm_DependenceEntropy were the nodes. The accuracy of the decision tree model was 0.889 with the AUC of 0.878.

**Figure 3:**
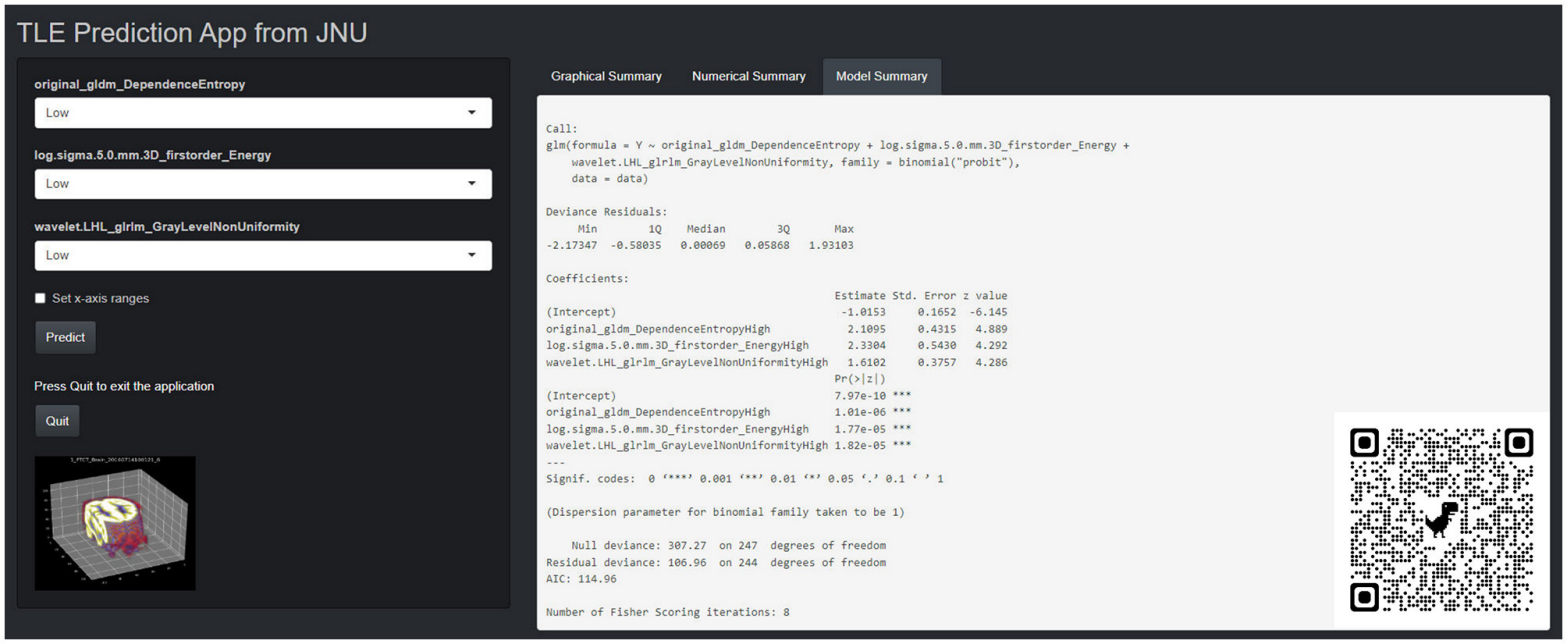
The corresponding online website version (https://wane199.shinyapps.io/TLE_DynNom/), showing the details of the LR model and the quick response code pasted on the bottom right corner of the screenshot.

**Figure 4:**
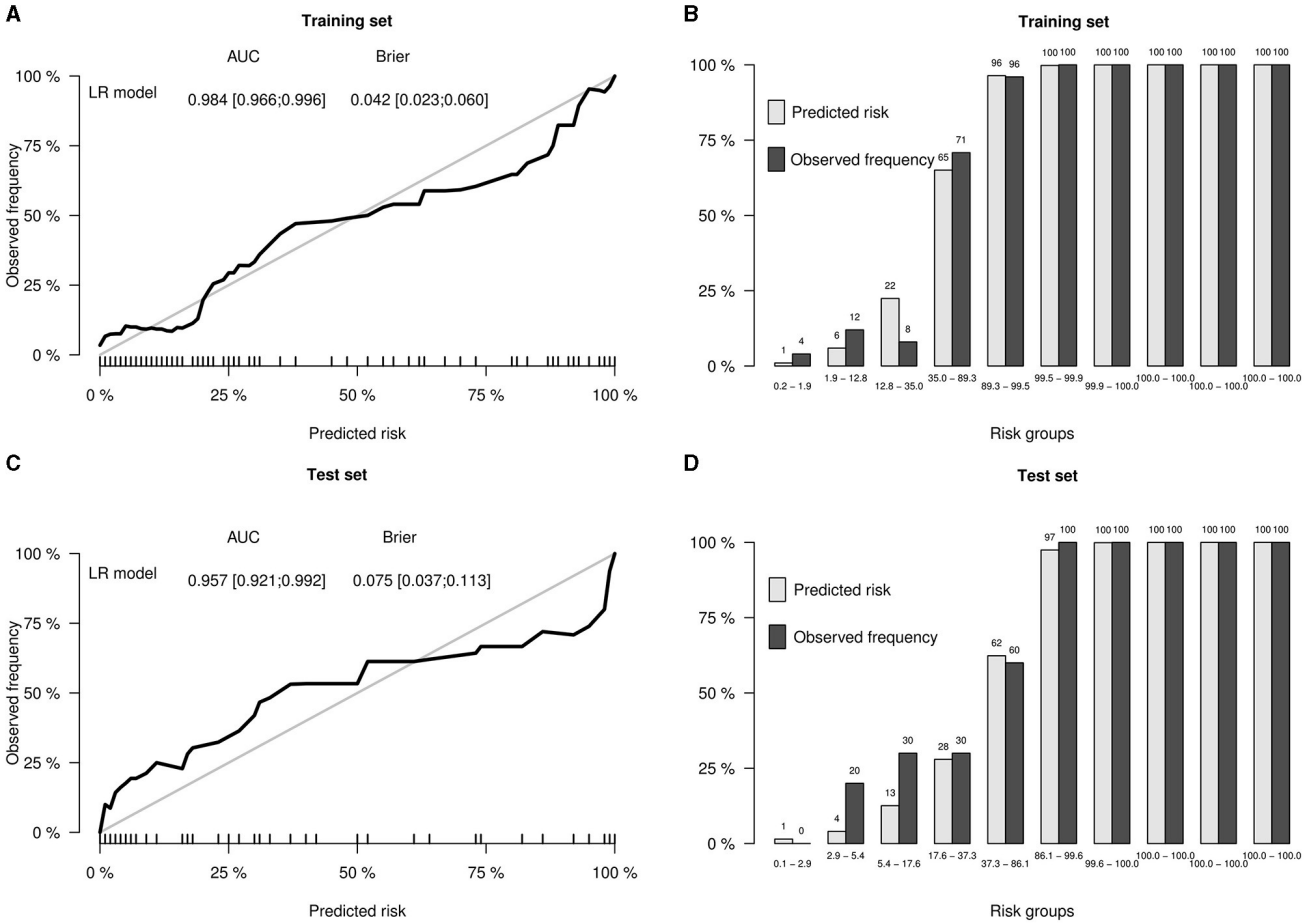
Performance of the LR model in the training and test sets. **(A)** Calibration curve of the training set, showing the AUC and the Brier score of the LR model in the training set. **(B)** Bar plots by risk groups to show calibration in the training set. **(C)** Calibration curve of the test set, showing the AUC and the Brier score of the LR model in the test set. **(D)** Bar plots by risk groups to show calibration in the test set.

This PET radiomics calculation was transformed into the “TLE Prediction App from JNU” online calculator (https://wane199.shinyapps.io/TLE_Classification/), as shown in Figure 3. The classification performance of the LR model in the training and the test sets is shown in Figure 4. The calibration curves indicated that this diagnostic nomogram exhibited good calibration.

## Discussion

In our study, we successfully extracted 1,132 radiomics features based on PET images and selected seven features using both Boruta and LASSO regression to generate ML models for identifying the candidate patients of TLE. The performance of 11 algorithms of model construction was compared, and the LR model was chosen to be the optimal method with the highest AUC. To optimize the LR model, Akaike Information Criterion (AIC) in a stepwise algorithm was used to further select the best three radiomics features, namely original_gldm_DependenceEntropy, log.sigma.5.0.mm.3D_firstorder_Energy, and wavelet.LHL_glrlm_GrayLevelNonUniformity. Extracted from the original image, original_gldm_DependenceEntropy is a feature based on a Gray Level Dependence Matrix (GLDM), which quantifies gray level dependencies in an image. DependenceEntropy is a measure of the randomness/variability in the gray level dependency defined as the number of connected voxels within the distance that is dependent on the center voxel. log.sigma.5.0.mm.3D_firstorder_Energy extracted from the image processed by Laplacian of Gaussian (log) filter with 5.0mm sigma is a measure of the magnitude of voxel values in this image. After the PET image had been managed with a wavelet filter combined with three dimensions, such as low, high, and low, wavelet.LHL_glrlm_GrayLevelNonUniformity could be extracted and represent the similarity of gray-level intensity values in the image, where a lower GrayLevelNonUniformity value correlates with a greater similarity in intensity values. Eventually, a tuned LR model based on three optimal PET radiomics features showed promising results in independent training and test cohorts, with an AUC of 0.981 and 0.957, respectively, for distinguishing TLE patients. Our study shows that PET-based radiomics can be useful biomarkers for identifying TLE patients.

Previous studies have shown that ^18^F-FDG PET is an available non-invasive method to complementally assist the diagnosis and prognosis prediction of epilepsy; it can also help with the intracranial electrode placement and potentially decrease the amounts of invasive EEG tests that need to be conducted (24–26). However, these studies were also focused on single conventional parameters. In such conditions, surgery is the most vital treatment for medication-refractory temporal lobe epilepsy, and ^18^F-FDG PET has important added value for the surgery decision-making in TLE because of the increased predictive values of MRI and video-EEG monitoring in combination with ^18^F-FDG PET.^18^F-FDG PET seemed especially valuable when MRI findings were negative or not concordant with EEG findings (20, 27–29). A meta-analysis compared the performance of ^18^F-FDG PET imaging with the traditional visual method for the localization of the epileptogenic zone in patients with epilepsy. Considering EEG or surgical outcomes as the gold standard, ^18^F-FDG PET demonstrated an overall sensitivity of 0.66 (95% CI: 0.58–0.73) and a specificity of 0.71 (95% CI: 0.63–0.78), with an AUC of 0.71 (30). Quantification of ^18^F-FDG PET might be helpful to improve the performance for epilepsy diagnosis and the localization of the epileptogenic zone. Wang et al. (31) compared the concordance rates with the gold standard evaluated by the visual assessment (40%), statistical parametric mapping (SPM, 83%), and three-dimensional stereotactic surface projection (3D-SSP,71%). The results showed that both SPM and 3D-SSP can improve the detection rate of the epileptic focus compared to the visual assessment, which demonstrated the great advantage of quantification.

Radiomics is a newly developing medical image analysis method with high-throughput extraction of quantitative features and automated quantification of radiographic phenotypes (32). Although the pathophysiology of epilepsy remains poorly understood, dysfunction of cerebral energy metabolism, neuronal loss, and reduction of synaptic activity may be involved, and PET is a direct reflection of cerebral energy metabolism and has been reported to partially reflect the reduction of synaptic activity (33). Radiomics features, especially high-order features, capture the spatial variation in PET signal intensity that may reflect the underlying pathophysiology, which may explain our observation. For repeatability in the present study, we combined the Boruta algorithm and LASSO regression to select radiomics features, resulting in the creation of a more generalized and stable classifier that is robust against the idiosyncrasies of the training data. In addition, a possible extension of this study can be the application of AAL atlas-derived segmentation procedure that allows for automated and accurate determination of ROIs, which would be especially useful for the segmentation of brain regions. Our study shows that using PET radiomics-based ML models can identify TLE patients because of high AUCs ranging from 0.878 to 0.984 in 11 various traditional machine learning algorithms (34). The logistic regression model, which is an efficient and powerful way to assess independent variable contributions to a binary outcome, was the final choice to distinguish TLE patients. Logistic regression can iteratively calculate the strongest linear combination of variables with the greatest probability of detecting the observed outcome (35). Identifying whether the participants are TLE patients or not is the objective of our study, which is a typical binary classification. This difference may explain why the LR model is the prime choice for the identification of TLE patients. The decision tree modeling is a non-parametric supervised learning algorithm, which is utilized for the classification task and is easy to explain visually. When analyzing our data using decision tree modeling, log.sigma.5.0.mm.3D_firstorder_Mean was the root node for classification, while wavelet.LHL_glrlm_GrayLevelNonUniformity and original_gldm_DependenceEntropy were selected by the LR model as internal nodes. These important PET radiomics features were subjected to bi-validation using various classical ML algorithms (Supplementary Figures S2, S3). Compared with decision tree modeling, logistic regression had a higher AUC value, which was more suitable for distinguishing TLE patients. In addition, an online web application to aid the diagnosis based on FDG PET data was developed according to our LR modeling results (https://wane199.shinyapps.io/TLE_Classification/). It could be used at different epilepsy centers, which can further validate the performance of our algorithm.

Our study has several limitations. First, the present investigation constitutes a retrospective analysis conducted solely within a singular healthcare facility, featuring a comparatively modest cohort size. Further investigations encompassing a broader dataset and external verification are essential to facilitate an enhanced evaluation. Second, we only extracted the radiomics feature in the temporal lobe region, so other important regions such as the hippocampus and the para-hippocampus should be investigated in future studies. Third, PET image feature selection still needs further optimization using deep learning techniques to ensure robustness and reproducibility. Future work should analyze the effect of PET radiomics-based ML models to characterize TLE from other centers, by extension, other epilepsy types. Fourth, our study focused on the classification performance of PET imaging independently. Therefore, we identified the TLE patients based only on single PET imaging, while clinical characteristics and other modal information of each patient was not involved. Further studies including more observations should be performed within multimodal data fusion approach.

## Conclusion

The findings from the current study provide proof of the potential performance of PET imaging in TLE diagnosis. In this study, we provided a convenient LR machine learning model based on PET radiomics features for TLE classification, which might be a promising technology for diagnosing TLE patients.

## Data availability statement

The raw data supporting the conclusions of this article will be made available by the authors, without undue reservation.

## Ethics statement

The studies involving humans were approved by the First Affiliated Hospital of Jinan University. The studies were conducted in accordance with the local legislation and institutional requirements. Written informed consent for participation was not required from the participants or the participants' legal guardians/next of kin because as this was a retrospective study, formal consent was not obtained.

## Author contributions

KL: Conceptualization, Data curation, Formal analysis, Validation, Writing – original draft. HW: Conceptualization, Formal analysis, Methodology, Writing – review & editing. YJ: Methodology, Validation, Writing – review & editing. CD: Data curation, Supervision, Writing – review & editing. HZ: Data curation, Writing – review & editing. BW: Data curation, Validation, Writing – review & editing. YT: Methodology, Visualization, Writing – review & editing. JG: Conceptualization, Data curation, Writing – review & editing. WY: Data curation, Writing – review & editing. YH: Investigation, Methodology, Writing – review & editing. QG: Conceptualization, Data curation, Investigation, Writing – review & editing. HX: Funding acquisition, Methodology, Resources, Validation, Writing – review & editing.
