# Supplementary material for: Machine learning techniques based on 18F-FDG PET radiomics features of temporal regions for the classification of temporal lobe epilepsy patients from healthy controls
Source: Front Neurol. 2024 Apr 9;15:1377538. doi: 10.3389/fneur.2024.1377538 (PMC11035742; doi:10.3389/fneur.2024.1377538)
Supplement: Supplementary file 1 [file Table_1.docx]

**Supplementary materials for Kai Liao et al.**

# Machine learning techniques based on ^18^F-FDG PET radiomics features of temporal regions for classification of temporal lobe epilepsy patients from healthy controls


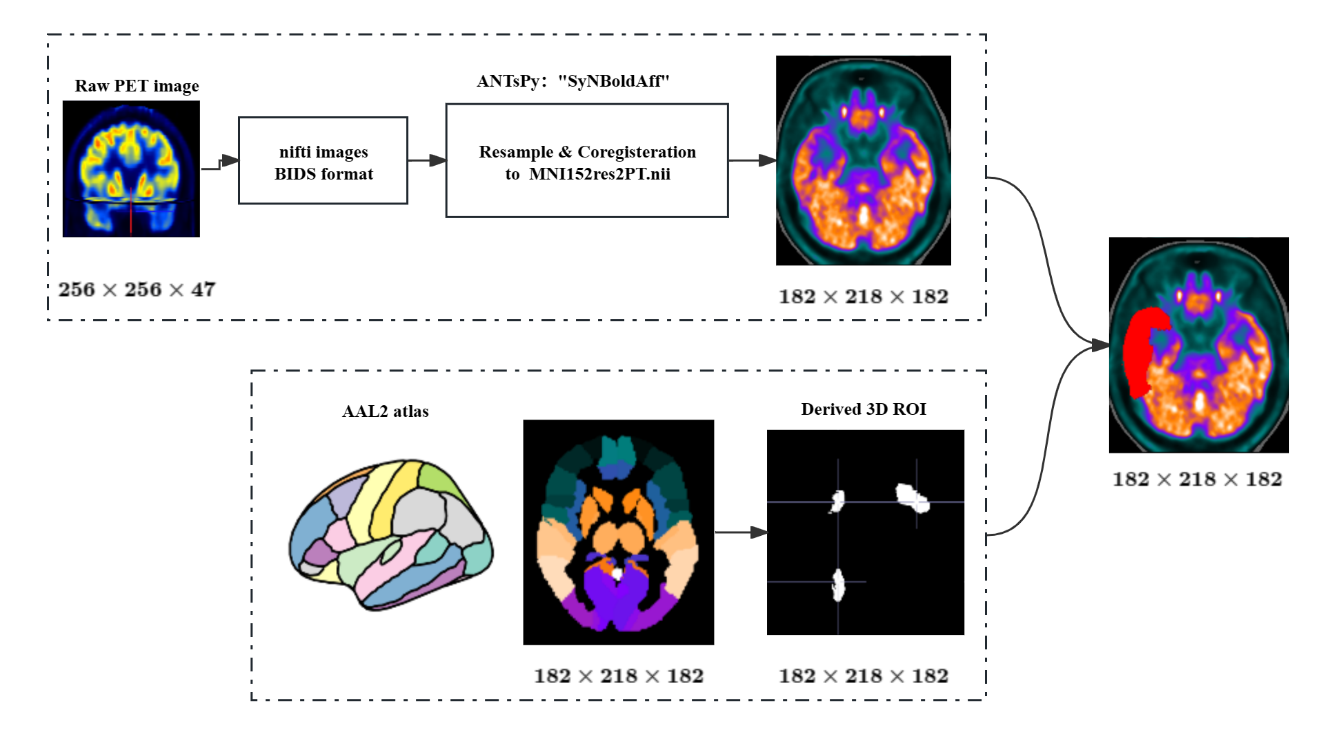
***Figure S1:*** The preprocessing schematic diagram for PET images.

Boruta is an all relevant feature selection wrapper algorithm, capable of working with any classification method that output variable importance measure (VIM); by default, Boruta uses Random Forest (1). The method performs a top-down search for relevant features by comparing original attributes' importance with importance achievable at random, estimated using their permuted copies, and progressively eliminating irrelevant features to stabilise that test.


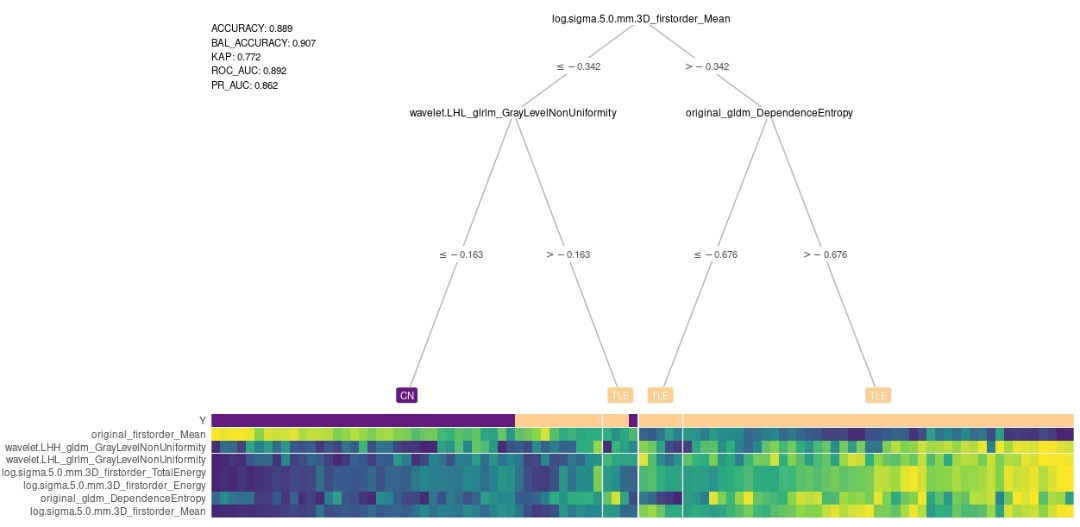


***Figure S2:*** The result of decision tree model using the 7 radiomics features selected by LASSO and Boruta algorithms.

Figure S2 displayed an interpretable decision tree visualization by integrating a heatmap at its terminal nodes (2). We created interpretable decision tree visualizations with the final 7 PET radiomics features represented as a heatmap at the tree's leaf nodes (original_firstorder_Mean, original_gldm_DependenceEntropy, log.sigma.5.0.mm.3D_firstorder_Energy, log.sigma.5.0.mm.3D_firstorder_Mean, log.sigma.5.0.mm.3D_firstorder_TotalEnergy, wavelet.LHL_glrlm_GrayLevelNonUniformity, wavelet.LHH_gldm_GrayLevelNonUniformity).


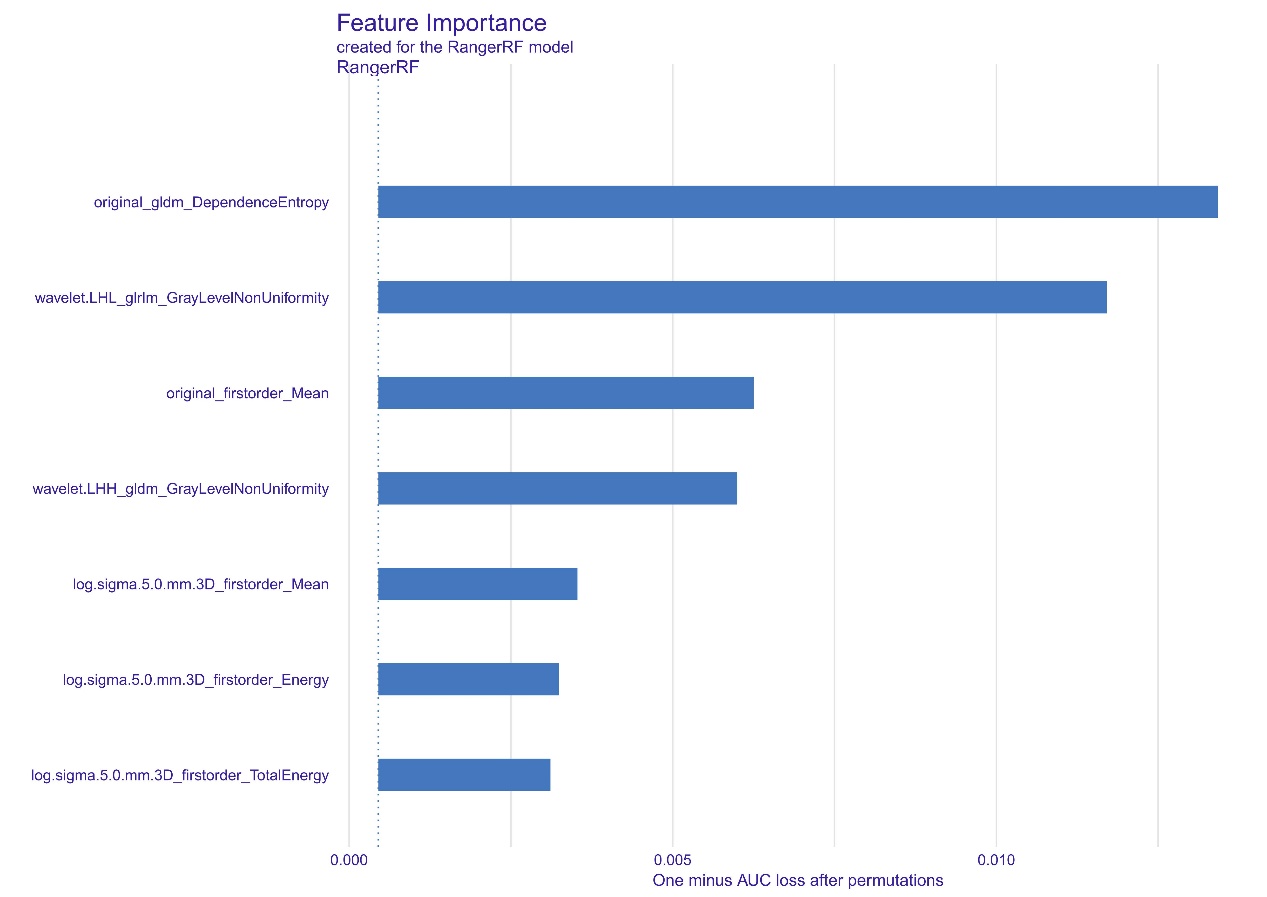


***Figure S3:*** Means (over 10 permutations) of permutation-based variable-importance measures for the explanatory variables included in the random forest model for our PET radiomics data using 1-AUC as the loss function.

Figure S3 showed the variable importance as change in loss function after variable permutations in our PET radiomics dataset (3). We used the area under the ROC curve (AUC) as the model performance measure. The plot in Figure S3 suggests that the most important variable in the model is original_gldm_DependenceEntropy. This agrees with the conclusions drawn in the exploratory analysis presented in table3. The next two important variables are wavelet.LHL_glrlm_GrayLevelNonUniformity, and original_firstorder_Mean.

***Table S1:*** The detailed setting of optimized hyperparameters in each 11 trained ML model.

| **Tuned model** | **Optimized hyperparameters (via PyCaret library of Python 3)** |
| --- | --- |
| **Logistic Regression** | LogisticRegression(C=1.0,class_weight=None,dual=False,fit_intercept=True, intercept_scaling=1,l1_ratio=None,max_iter=1000,multi_class='auto', n_jobs=None,penalty='l2',random_state=123, solver='lbfgs', tol=0.0001, verbose=0,warm_start=False) |
| **Naive Bayes** | GaussianNB(priors=None, var_smoothing=1e-09) |
| **Linear Discriminant Analysis** | LinearDiscriminantAnalysis(covariance_estimator=None,n_components=None, priors=None, shrinkage=None, solver='svd',store_covariance=False, tol=0.0001) |
| **Random Forest Classifier** | RandomForestClassifier(bootstrap=True,ccp_alpha=0.0, class_weight=None,  criterion='gini', max_depth=None, max_features='sqrt',max_leaf_nodes=None, max_samples=None,min_impurity_decrease=0.0, min_samples_leaf=1,  min_samples_split=2, min_weight_fraction_leaf=0.0, n_estimators=100, n_jobs=-1, oob_score=False,random_state=123, verbose=0, warm_start=False) |
| **Extra Trees Classifier** | ExtraTreesClassifier(bootstrap=False,ccp_alpha=0.0, class_weight=None,criterion='gini', max_depth=None,  max_features='sqrt',max_leaf_nodes=None, max_samples=None,  min_impurity_decrease=0.0,min_samples_leaf=1,min_samples_split=2, min_weight_fraction_leaf=0.0,n_estimators=100, n_jobs=-1,  oob_score=False,random_state=123, verbose=0, warm_start=False) |
| **Gradient Boosting Classifier** | GradientBoostingClassifier(ccp_alpha=0.0,criterion='friedman_mse', init=None,learning_rate=0.01, loss='log_loss', max_depth=7,max_features='sqrt', max_leaf_nodes=None,min_impurity_decrease=0.05, min_samples_leaf=2,  min_samples_split=2, min_weight_fraction_leaf=0.0,n_estimators=140, n_iter_no_change=None,random_state=123,subsample=0.35, tol=0.0001,  validation_fraction=0.1, verbose=0,warm_start=False) |
| **Light Gradient Boosting Machine** | LGBMClassifier(bagging_fraction=0.7,bagging_freq=6, boosting_type='gbdt',  class_weight=None, colsample_bytree=1.0, feature_fraction=0.5, subsample=1.0,  importance_type='split', n_jobs=-1, learning_rate=0.1, max_depth=-1,min_child_samples=66, min_child_weight=0.001, min_split_gain=0.4,  n_estimators=90, num_leaves=90, objective=None,random_state=123,  reg_alpha=0.0005, reg_lambda=0.1,silent='warn', subsample_for_bin=200000,  subsample_freq=0) |
| **K Neighbors Classifier** | KNeighborsClassifier(algorithm='auto', leaf_size=30, metric='manhattan',  metric_params=None, n_jobs=-1, n_neighbors=13, p=2,weights='distance') |
| **Ada Boost Classifier** | AdaBoostClassifier(algorithm='SAMME',base_estimator=None, learning_rate=0.4,n_estimators=60, random_state=123) |
| **Quadratic Discriminant Analysis** | QuadraticDiscriminantAnalysis(priors=None, reg_param=0.0,store_covariance=False, tol=0.0001) |
| **Decision Tree Classifier** | DecisionTreeClassifier(ccp_alpha=0.0,class_weight=None, random_state=123, criterion='gini',max_depth=16,max_features='log2', max_leaf_nodes=None,  min_impurity_decrease=0.0002, min_samples_leaf=3,min_samples_split=2, min_weight_fraction_leaf=0.0, splitter='best') |


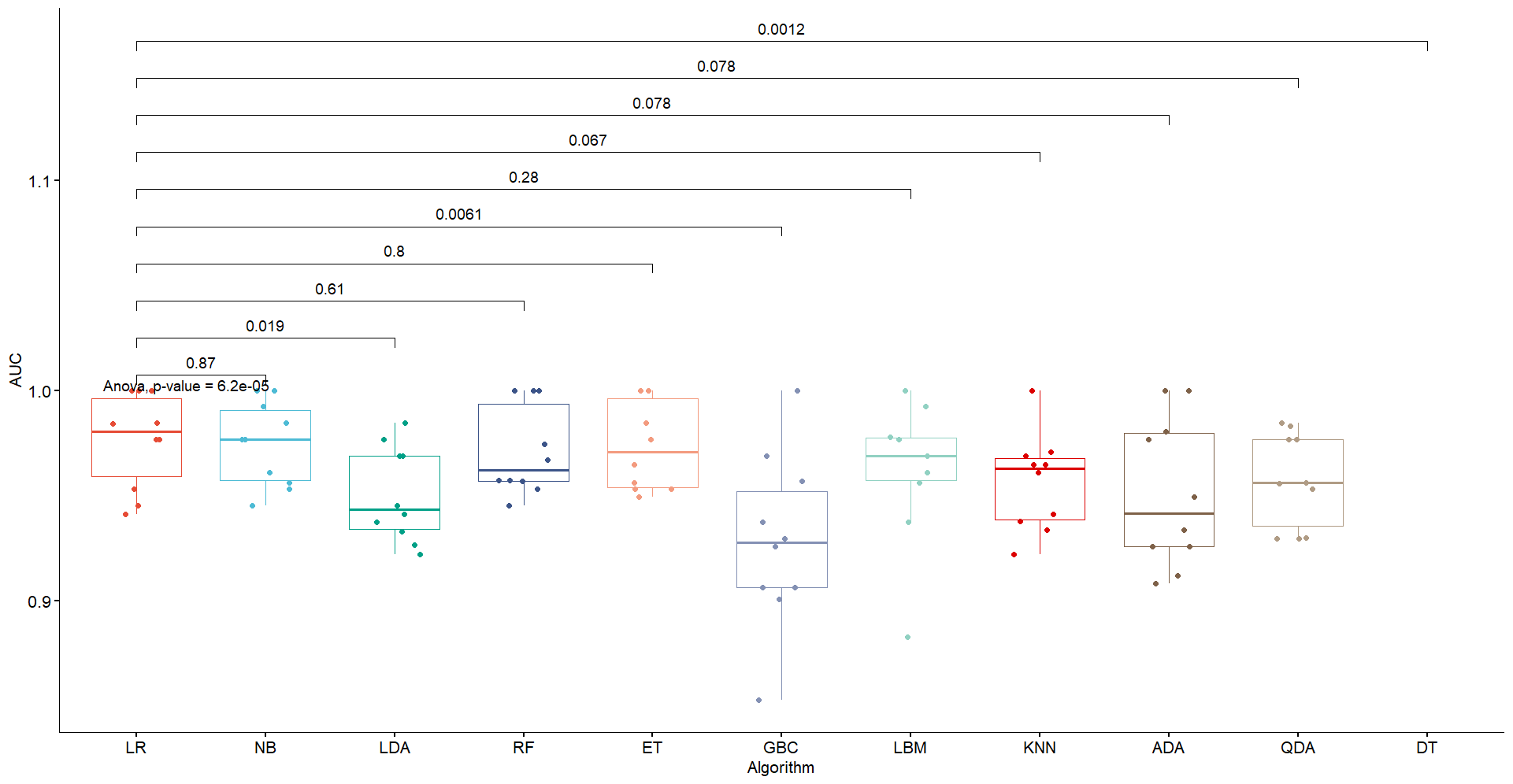


***Figure S4:*** The comparison of the mean 10-fold cross-validation AUC between the LR model and other 10 ML algorithms. A one-way ANOVA revealed that there was a statistically significant difference in the mean AUC between at least two ML algorithms (*p* < 0.001). The two sample t-test showed that there was a significant difference in the mean AUC between LR model and LDA (or GBC, DT algorithm) algorithm (*p* < 0.05), but not a significant difference in the mean AUC between LR model and NB (or RF, ET, LBM, KNN, ADA, GBA algorithm) algorithm (*p* > 0.05).

We performed the one-way ANOVA and two sample t-test to compare the 10-fold cross-validation AUC between the LR model and other 10 ML algorithms. The result showed that there was a statistically significant difference in the mean AUC between at least two ML algorithms (*p* < 0.001). And there was a significant difference in the mean AUC between LR model and LDA (or GBC, DT algorithm) algorithm (*p* < 0.05).

**References**

1. Kursa MB, Rudnicki WR. Feature selection with the Boruta package. *Journal of statistical software* (2010) 36:1–13. doi: 10.18637/jss.v036.i11

2. Le T, Moore J. treeheatr: Heatmap-integrated decision tree visualizations. [manual]. (2020). https://CRAN.R-project.org/package=treeheatr

3. Biecek P. DALEX: Explainers for complex predictive models in R. *Journal of Machine Learning Research* (2018) 19:1–5.
